# Nonlinear profile order for 3D hybrid radial acquisition applied to self-gated free-breathing cardiac cine MRI

**DOI:** 10.1186/1532-429X-18-S1-P23

**Published:** 2016-01-27

**Authors:** Yanchun Zhu, Shuo Li, Jie Yang, Rongmao Li, Zhicheng Zhang, Shaode Yu, Yaoqin Xie

**Affiliations:** grid.458489.c0000000104837922Institute of Biomedical and Health Engineering, Shenzhen Institutes of Advanced Technology, Shenzhen, China

## Background

Self-gated free-breathing 3D cardiac cine MRI has been widely introduced, which provides similar image quality and functional measurements as breath-hold technique [1–3]. In self-gated free-breathing cardiac cine MRI, respiratory and cardiac motions are unpredictable during the acquisition especially in retrospective reconstruction. Therefore, nonuniform k-space distribution is a major problem in retrospective self-gated reconstruction.

## Methods

αA previously developed hybrid radial sampling [2, 3] was adapted for 3D cardiac cine imaging. A nonlinear profile order with variant azimuthal increment was provided as a substitution for golden ratio based profile order. The projection angle is incremented nonlinearly based on formula θ=α × π × P^β^, where θ is projection angle of the profile number P, and α and β are variable parameters. Optimal parameters in the nonlinear formula were chosen based on the simulations. These two profile orders were compared among k-space distribution, phantom and human image results. Nine normal subjects (32 ± 7 yo, 7 male) were imaged by both nonlinear profile order and golden-ratio based scheme. Image quality including images sharpness, contrast, blood SNR and blood-to-myocardium CNR obtained with both methods were compared. The study was approved by the local institutional review board, and written informed consent was obtained from all subjects.

## Results

**Figure 1:**
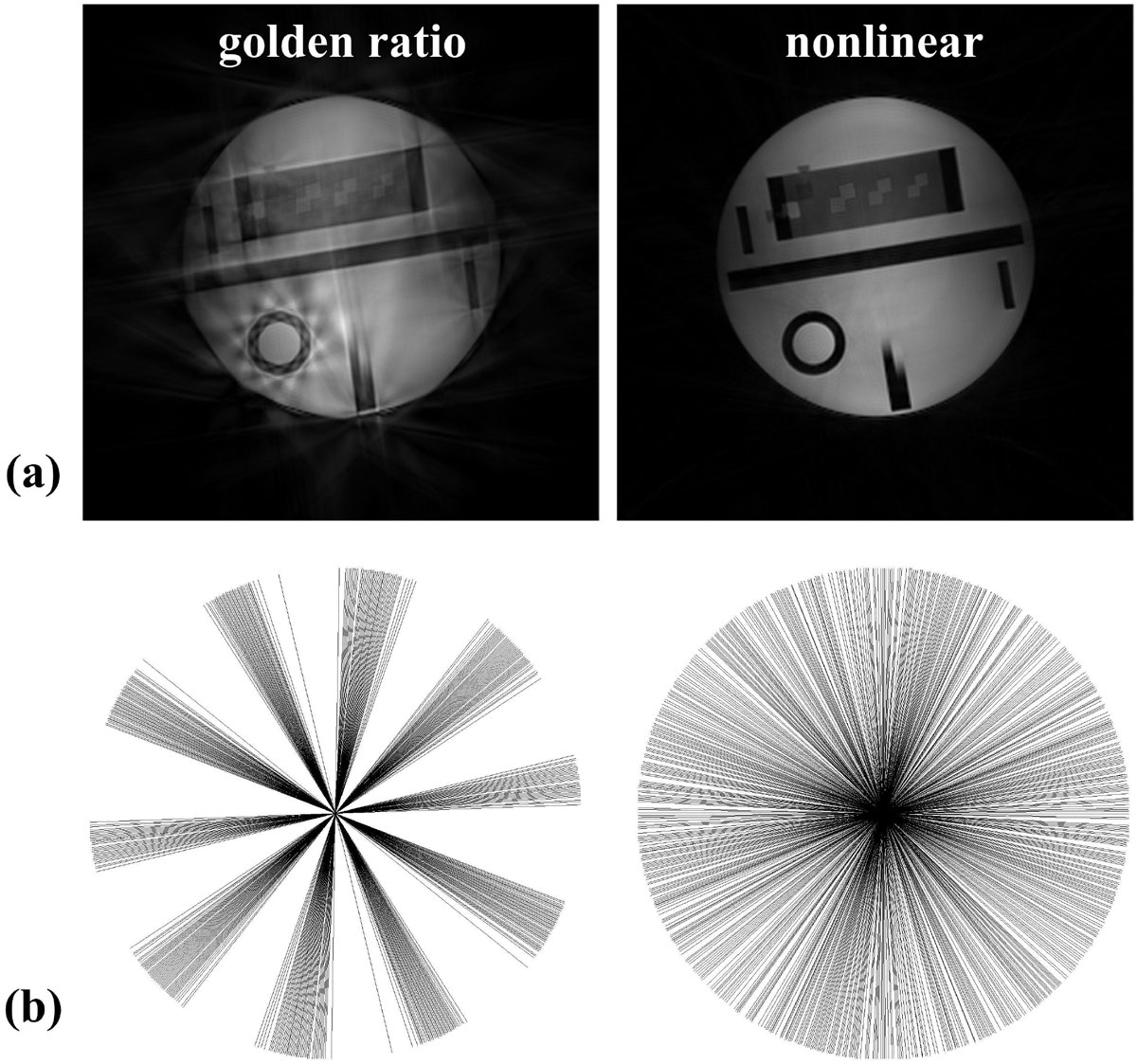
**The reconstructed phantom images (a) and profile distribution (b) based on the same self-gating signals**.

Optimized α and β were 1.66 and 0.28 from simulation where high sampling efficiency [4] was obtained. Figure 1 shows the reconstructed phantom images and profile distributions based on the self-gating signals. Nearly 540 profiles were used in the reconstruction including shared neighboring profiles. In comparison, obvious streak artifact appears in the golden ratio based profile order, which is caused by non-uniform distribution of profiles. However, more uniform distribution is generated based on nonlinear profile order. The SE of golden ratio and nonlinear profile order are 0.83 and 0.24 respectively. Image reconstruction was successfully in nine subjects. Table 1 shows the image quality comparison of mid-ventricular slices obtained during end-diastolic and end-systolic cardiac phases from all volunteers. Comparable image quality are observed in blood SNR, myocardium-blood CNR, myocardium contrast, and image sharpness. The differences were not statistically significant (P > 0.05).

**Table 1:** Image quality measurements of the two methods (N = 9).

	SNR_Blood_	CNR_Blood-Myocardium_	Contrast_Blood-Myocardium_	Image Sharpness(mm^-1)^
Golden Ratio Diastole	53.5 ± 25.3	22.0 ± 14.8	24.7 ± 6.9	0.21 ± 0.04
Nonlinear Diastole	54.4 ± 28.5	22.2 ± 17.0	24.9 ± 9.9	0.24 ± 0.03
P-Value	0.767	0.678	0.767	0.110
Golden Ratio Systole	54.4 ± 18.6	22.1 ± 10.7	24.7 ± 6.8	0.22 ± 0.05
Nonlinear Systole	51.9 ± 21.0	22.2 ± 13.7	26.2 ± 11.4	0.26 ± 0.07
P-Value	0.173	0.678	0.515	0.086

## Conclusions

Approximate uniform distribution was obtained based on nonlinear profile order among persons with different heart and breath patterns. Nonlinear profile order can provide more stable profile distributions and fewer streaking artifacts. In conclusion, the nonlinear based profile order was proved to be insensitive to different motion patterns, and more useful in retrospective reconstruction.
